# Epidemiology of Sexually Transmitted Infections among Human Immunodeficiency Virus Positive United States Military Personnel

**DOI:** 10.1155/2013/610258

**Published:** 2013-04-21

**Authors:** Jeff S. Tzeng, Leslie L. Clark, Eric C. Garges, Jean Lin Otto

**Affiliations:** ^1^Division of Preventive Medicine, Walter Reed Army Institute of Research, 503 Robert Grant Avenue, Silver Spring, MD 20910, USA; ^2^Armed Forces Health Surveillance Center, 11800 Tech Road, Suite 220, Silver Spring, MD 20904, USA; ^3^Henry M. Jackson Foundation for the Advancement of Military Medicine, Bethesda, MD 20817, USA

## Abstract

*Background*. Minimal data exist that describe the epidemiology of sexually transmitted infections (STI) in human immunodeficiency virus (HIV) positive populations across the pre- and post-diagnosis periods for HIV. *Purpose*. The purpose of this study was to identify and describe the epidemiology of gonorrhea, chlamydia, syphilis, herpes simplex virus, and human papillomavirus in an HIV-positive population. *Methods*. All 1,961 HIV seropositive United States active duty military personnel from 2000–2010 were identified. STI diagnoses relative to HIV diagnosis from 1995, which was the earliest electronic medical record available, to 2010 were examined. *Results*. The incidence diagnosis rates of STI generally increased during the period leading up to eventual HIV diagnosis. The rates of STI during the post-HIV diagnosis period fluctuated, but remained elevated compared to pre-HIV diagnosis period. Approximately 45%–69% with an STI in the HIV seropositive military population were diagnosed with their first STI greater than one year after their HIV diagnosis. Of those who were diagnosed with an STI in the post-HIV diagnosis period, 70.6% had one STI diagnosis, 23.5% had two STI diagnoses, and 5.8% had three or more STI diagnoses. *Conclusions*. Despite aggressive counseling, high-risk sexual behavior continues to occur in the HIV-positive military population.

## 1. Introduction

In the United States (US), an estimated 56,300 new human immunodeficiency virus (HIV) infections were diagnosed in 2006, with an incidence rate of 22.8 infections per 100,000 people [1]. During that same year, there were 196 new HIV-1 diagnoses in the active duty US military with an incidence rate of 18.3 per 100,000 tested [2].

Increasing numbers of lifetime sexual partners, decreased condom usage, and prior sexually transmitted infections (STI) have all been reported to increase the risk of HIV transmission [3–5]. With the advent of effective antiretroviral medications, there has been a reduced risk of HIV transmission to HIV-negative partners in some situations (i.e., preexposure prophylaxis) [6]. Increased unsafe behavioral risk factors have been noted in some HIV-positive populations, with approximately 32–39% of HIV-positive people engaging in unprotected intercourse [7]. This not only increases the risk of transmitting HIV to an HIV-negative sexual partner, but also places the HIV-positive partner at risk of acquiring an STI.

In 1985, the US military began conducting mandatory routine screening for HIV-1 antibodies and at least biennially (once every 2 years) since 2004. The epidemiology has been well described [2, 8], but routine STI screening is limited to laboratory-based screening for cervical human papillomavirus (HPV), gonorrhea, and chlamydia during required routine women's health encounters and can differ based on age and risk factors. Screening in some populations with perceived higher risk of STI acquisition is often performed at the discretion of the health care provider [8].

There are very few studies that describe the distribution of STI in the HIV-positive military population [9–11]. The epidemiology of STIs in an HIV-positive population is important to understand, not only to evaluate risk of new infections, but also to explore the impact of high risk sexual practices in this population. To study the relationship between STIs and HIV, a retrospective study of HIV-positive active duty US military personnel was performed to describe the distribution of chlamydia, gonorrhea, syphilis, HPV, and herpes simplex virus (HSV) incident diagnosis during the pre- and post-HIV diagnosis periods.

## 2. Methods

### 2.1. Study Population and Data Sources

The surveillance population included all active duty military personnel of the US Army, US Navy, US Air Force, US Marine Corps, and US Coast Guard who were HIV-1 antibody seropositive from January 1, 2000, to December 31, 2010. HIV-1 positivity was defined as one positive serological screening test such as enzyme-linked immunosorbent assay and another from a confirmatory test such as western blot. The date of the earliest positive serological test was utilized as the reference date for HIV-1 positivity for that individual.

We examined all electronic medical record data for this HIV-1 positive cohort available in the Defense Medical Surveillance System (DMSS) retrospectively from December 31, 2010, to January 1, 1995 (this is the earliest date for availability of the electronic medical records used for this analysis). The DMSS is a database managed by the Armed Forces Health Surveillance Center containing records documenting all ambulatory encounters and hospitalizations of active component members of the US military and other historical and current information on medical events (e.g., reportable diseases, HIV tests, immunizations, and other health risk appraisals). These records include all medical care provided in fixed military facilities and medical care provided in civilian facilities that is reimbursed through the Military Health System. 

### 2.2. Case Definitions

The DMSS was used to identify all medical encounters occurring in a military treatment facility or reimbursed through the Military Health System within the HIV-positive population that contained diagnostic codes (*ICD-9-CM*) for chlamydia (*099.41, 099.5*), gonorrhea (*98*), syphilis (*091–097*), HSV (*54*), and HPV (*078.1, 079.4, 795.05, 795.09, 795.15, 796.75, and 796.79*). A positive STI case was defined as one inpatient or one outpatient visit with the specified ICD-9 CM code in the first or second diagnostic position. An individual was eligible to be an incident case once per 30-day period for chlamydia and gonorrhea, once per 13-month period for syphilis, and once per lifetime for HSV and HPV. 

For each unique HIV-positive individual, we determined incident STI case date(s), HIV seropositive reference date, time elapsed from incident STI date to HIV reference date, time elapsed from HIV reference date to first and subsequent incident STI date, time elapsed from entrance into active duty service to HIV reference date, and time from HIV reference date to the end of US active duty military service or December 31, 2010, whichever occurred earliest.

### 2.3. Data Analysis

Incidence rates were calculated by summing incident STI cases occurring before the HIV reference date and dividing by the total number of person-years prior to the HIV reference date. This pre-HIV diagnostic window of observation was limited by time in the military prior to active duty and was censored by the earliest available electronic medical record on file in DMSS (January 1, 1995). This was repeated similarly to calculate incident STI cases occurring after HIV reference date. The post-HIV diagnostic person time was limited by time of military separation or December 31, 2010, whichever occurred first. The time from HIV reference date to the first and subsequent incident STI case(s) was placed into one of seven groups: 0–7 days, 8–30 days, 31–60 days, 61–90 days, 91–180 days, 181–365 days, and >365 days. Proportions were calculated by summing the frequency of first STI diagnosis post-HIV within each date category and dividing by the total frequency of first time incident cases for the specific STI.

## 3. Results

### 3.1. Study Population

We identified a total of 1,961 HIV-1 positive active duty military personnel during the period from 2000 to 2010 (Table 1). The majority of HIV-1 positive cases were male (96.8%), Black non-Hispanic, (52.5%), with a high school education only (66.9%) and single at the time of HIV-1 diagnosis (66%). The 20–24-year-old age group comprised nearly 38% of HIV-1 positive cases and had the highest incidence rate (14.3 per 100,000 person-years). 

### 3.2. STI before and after HIV Diagnosis

**Figure 1:**
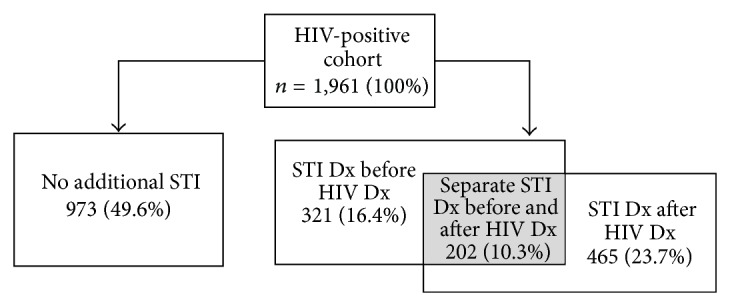
Proportions of sexually transmitted infection (STI) diagnosis made in human immunodeficiency virus (HIV) positive United States military cohort relative to HIV diagnosis (Dx). *n* = 1,961.

During the surveillance period, 988 (50%) of the HIV-positive military population were diagnosed with any STI: 321 with only STI before HIV, 465 with only STI after HIV, and 202 with any STI both before and after HIV diagnosis (Figure 1). Of the HIV-positive population who had any STIs before their HIV diagnosis, there were 182 incident diagnoses of gonorrhea from 144 unique individuals, representing an incidence rate of 1,721.5 cases per 100,000 pre-HIV person-years. There were 97 unique individuals who accounted for 119 incident diagnosis of chlamydia, representing an incidence rate of 1,125.6 cases per 100,000 pre-HIV person-years. HSV represented the smallest number of incident cases and unique individuals with 58 (548.6 cases per 100,000 pre-HIV person-years), while HPV represented the largest number with 158 incident cases and unique individuals (1,494.5 cases per 100,000 pre-HIV person-years). Black non-Hispanics represented the highest incidence rates of chlamydia, gonorrhea, and syphilis among those who had an STI before their eventual HIV diagnosis. Being single, 17–19-year-old age group or lack of a college education consistently showed the highest incidence rates for all STIs evaluated among those who had an STI before their eventual HIV diagnosis.

A total of 667 unique individuals were diagnosed with an STI after their HIV diagnosis. Of those who were diagnosed with any STIs in the post-HIV diagnosis period, 70.6% had one STI diagnosed, 23.5% had two STIs diagnosed, and 5.8% had three or more STIs diagnosed (Table 2). In this population, the proportion was highest among non-Hispanic Black, single people, enlisted rank, the 20–24-year-old age group and high school only educated personnel.

**Figure 2:**
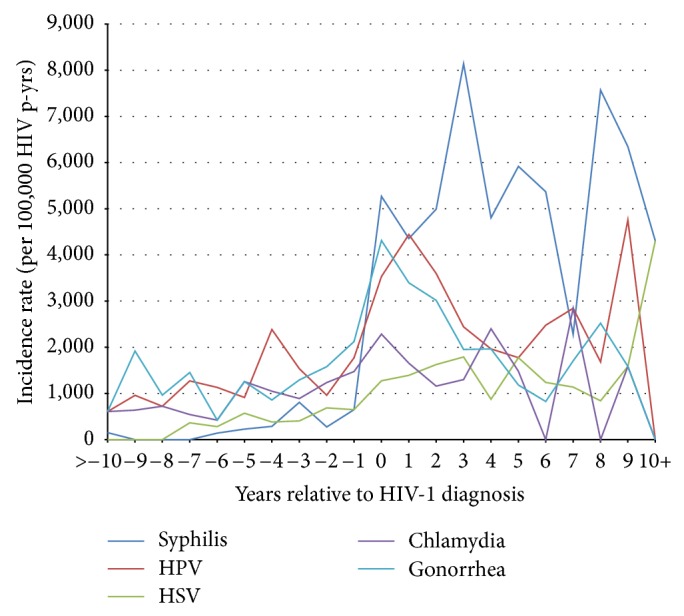
Incidence rates of sexually transmitted infections diagnoses relative to human immunodeficiency virus-1 (HIV-1) diagnosis by year in United States active duty military personnel.

There were 157 unique individuals who generated 186 incident cases of gonorrhea after their HIV diagnosis (Table 3). Syphilis accounted for the largest number of unique individuals (312) and incident cases (364) after their HIV diagnosis. Among those who had an STI after their HIV diagnosis, the 25–29-year-old age group showed the highest incidence rates for chlamydia, HSV, and gonorrhea. Individuals with syphilis had the lowest incidence rate of STIs examined in period before their HIV diagnosis, but had the highest incidence rate in the period after their HIV diagnosis (Figure 2). The incidence rate of gonorrhea generally increased during the 10-year period leading up to HIV diagnosis, but consistently decreased in the 10-year period after the HIV diagnosis.

**Figure 3:**
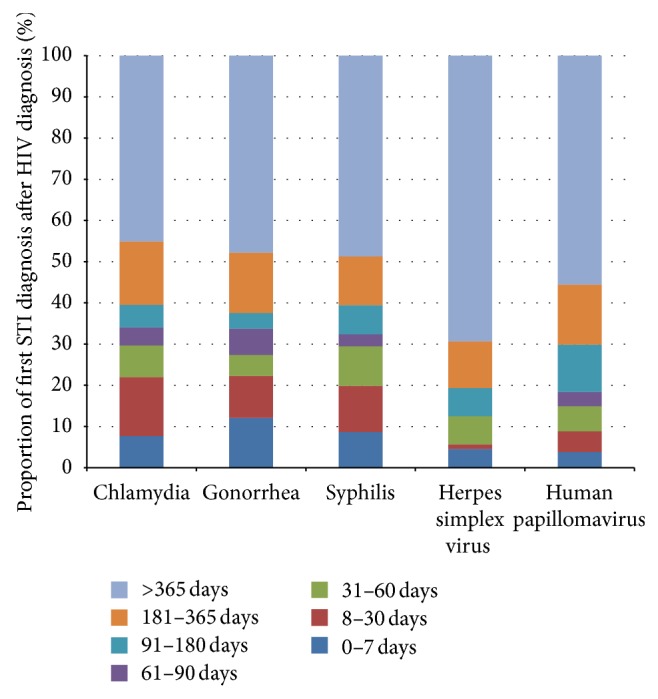
The distribution of human immunodeficiency virus (HIV) diagnosis by time to first sexually transmitted infection (STI) diagnosis in United States active duty military personnel.

Figure 3 represents the time elapsed from HIV diagnosis to the first STI diagnosis. Approximately 45%–69% with an STI in the HIV-positive military population were diagnosed with their first STI greater than a year after their HIV diagnosis. Conversely, 4%–12% were diagnosed with their first STI within 7 days of their HIV diagnosis.

## 4. Discussion

One of the most important findings of this study is the elapsed time from HIV diagnosis to first STI diagnosis. In our study population, approximately half of all initial STIs diagnosed among HIV-positive persons occurred one year or more after their HIV diagnosis. This appears to be consistent with some studies suggesting that while high-risk sexual behaviors decrease immediately after HIV diagnosis, there does not appear to be an elimination of these behaviors. The probability of high-risk behaviors increases as individuals are further from the time of their HIV diagnosis [12–14].

While the continuation of high-risk sexual activity after HIV diagnosis can be inferred from the results of this study, it is difficult to assess the exact magnitude of risk (i.e., frequency, nature of behavior) due to a variety of limitations in the available data. The peak period of STI incidence rates immediately before and after the HIV reference date is likely due to concurrent testing and diagnosis of HIV and STI. This is supported by the finding that 70% of those with an STI in the post-HIV diagnosis period have only one STI diagnosis, while 30% have two or more STI diagnoses.

The Center for Disease Control and Prevention (CDC) recommends annual screening for curable STI in sexually active HIV-positive persons [15]. Despite this recommendation, routine testing for STI in the nonmilitary HIV-positive population, especially in asymptomatic patients, remains low [16]. The US Army, US Navy, US Air Force, and US Coast Guard have similar rules and regulations regarding HIV testing, follow-up surveillance, and patient counseling. It is reasonable to expect that STI testing is consistent with the CDC guidance, but little data exists. All military branches do require HIV-positive active duty military personnel medical followup at a minimum of every six to twelve months; however, there is no mandate that STI screening occur at specified intervals [17–20].

The counseling that occurs when an active duty military personnel is notified of HIV seropositivity is explicit and uniform among the different military branches. HIV-positive military personnel who wish to continue on active duty must agree to key elements: notification of present and future sexual partners of HIV status, use of Food Drug Administration approved barrier protective devices when engaging in sexual activities after receiving consent from their partner, and notification of their military medical providers of any changes to health [17–19]. Failure to comply with these orders can result in disciplinary actions under the Uniform Code of Military Justice and/or separation from the military, which could result in loss of all medical care and benefits. HIV-positive military personnel may interpret these orders as a mandate for compulsory reporting of all medical symptoms that arise. This could lead to increased medical testing in general and STI testing specifically, introducing an additional surveillance bias.

Another limitation of this study is the exclusive use of ICD-9 code diagnoses to determine STI cases. Rather than being based on laboratory confirmation, STI cases are based on healthcare provider-assigned diagnostic codes entered into the electronic medical record of military personnel. Ideally, when an STI is suspected, laboratory testing results. However, some health care providers may find a clinical diagnosis sufficient for treatment purposes and not utilize the laboratory. The addition of nucleic acid testing (NAAT) in the early 2000's raised the baseline rate of chlamydia by increased screening in asymptomatic men because testing only required urine and not a urethral swab. It is not expected that this diagnostic bias would have much effect on the study data outside the additional diagnosis of chlamydia and to a lesser degree gonorrhea in males, due to the more symptomatic nature of gonorrhea. While the reliability of our ICD-9-based case definition in identifying actual cases of STIs is unknown, preliminary data demonstrate high correlation between ICD-9 code diagnoses and laboratory confirmation for gonorrhea, chlamydia, and syphilis. Analyses examine the percentage of STI reportable medical events that have a positive laboratory test: 80% for gonorrhea, 85% for Chlamydia, and 95% for syphilis (personal communication, Dr. Leslie Clark). Nonetheless, exclusive use of ICD-9 code diagnoses to determine STI cases likely results in an overestimation of STI cases in this study.

STI rates in this population may also be underestimated for several reasons. There are incentives for underreporting of STI-related symptoms due to disciplinary action that could be taken because of a perceived or real violation of these orders outlines previously. Additionally, HIV-positive military personnel may utilize resources that are not reimbursed through the military medical system, such as publically funded clinics or pay out of pocket for out of network medical care. This would not result in an STI diagnostic code that is captured by the DMSS database. Several studies have described additional barriers to reporting and seeking medical care for STIs that are equally applicable to this population, namely, stigma and perception of shame [21–23]. Another important reason pertains to the often asymptomatic nature of some viral STIs. These individuals would be unaware they have an STI and would likely not seek medical care.

As expected, the STI incidence rates of this study cohort in the pre-HIV diagnosis period were higher compared to the general active duty military population for chlamydia, gonorrhea, and syphilis. The rate of chlamydia in the general active duty military population (1,056.2 cases per 100,000 person-years) was similar to the rate of this cohort pre-HIV diagnosis (1,125.6 cases per 100,000 person-years) [8]. The incidence rate of gonorrhea in the pre-HIV diagnosis period (1,721.5 cases per 100,000 person-years) was over seven times higher compared to the general active duty military population (230.8 cases per 100,000 person-years). The incidence rate of syphilis was 18 times higher in the pre-HIV diagnosis period compared to the general active duty military population with 624.3 and 34.6 cases per 100,000 person years, respectively. The rates of HSV and HPV were lower in the general active duty military population compared to the pre-HIV diagnosis period. The association between syphilis and HIV is well described and was anticipated in this population, but the high gonorrhea incidence rate warrants further investigation [24, 25].

In conclusion, this study examined the relationship between STIs in a low HIV prevalence US military cohort before and after their HIV diagnosis. Overall, the remarkable finding that nearly half of the first STI diagnosis occurred greater than one year after HIV diagnosis warrants further investigation and indicates that high-risk sexual activity is continued to be practiced by this group of individuals.

## Figures and Tables

**Table 1:** Characteristics of human immunodeficiency virus-1 seropositive United States active duty military personnel, 2000–2010.

	2000		2001		2002		2003		2004		2005		2006		2007		2008		2009		2010		Total		
	No.	Rate (per 100,000 p-yrs)	No.	Rate (per 100,000 p-yrs)	No.	Rate (per 100,000 p-yrs)	No.	Rate (per 100,000 p-yrs)	No.	Rate (per 100,000 p-yrs)	No.	Rate (per 100,000 p-yrs)	No.	Rate (per 100,000 p-yrs)	No.	Rate (per 100,000 p-yrs)	No.	Rate (per 100,000 p-yrs)	No.	Rate (per 100,000 p-yrs)	No.	Rate (per 100.000 p-yrs)	No.	Rate (per 100.000 p-yrs)	Proportion (%)

	Sex																								

Male	142	11.9	166	13.9	158	13.0	157	12.7	163	13.2	162	13.4	166	13.9	156	13.0	206	16.9	204	16.5	219	17.5	1899	14.2	96.8
Female	8	4.0	10	4.9	8	3.8	4	1.9	5	2.3	2	1.0	4	2.0	6	3.0	2	1.0	4	1.9	9	4.3	62	2.7	3.2

	Age group																								

17–19	9	7.2	9	7.1	8	6.5	7	6.1	6	5.4	6	6.1	7	7.3	8	8.2	6	6.0	13	13.8	9	10.4	88	7.5	4.5
20–24	52	11.9	62	13.7	49	10.3	52	10.4	71	14.2	65	13.5	71	15.0	65	13.9	80	16.9	83	17.4	94	19.5	744	14.3	37.9
25–29	34	12.2	42	15.6	32	11.6	42	14.6	35	11.7	26	8.5	35	11.3	38	12.0	57	17.4	54	15.7	56	15.8	451	13.4	23.0
30–34	27	12.7	32	15.5	36	17.4	27	13.0	23	11.1	25	12.3	20	10.1	22	11.1	32	15.9	29	13.9	26	12.0	299	13.2	15.2
35–39	22	10.7	21	10.6	27	13.9	21	11.2	21	11.6	27	15.5	20	11.6	21	12.2	20	11.6	17	9.9	23	13.6	240	12.0	12.2
40–44	3	3.2	9	9.1	10	9.6	9	8.5	10	9.4	10	9.5	9	8.8	8	8.l	11	11.3	11	11.2	17	17.2	107	9.6	5.5
45–49	2	6.2	1	3.0	4	11.5	3	8.6	2	5.6	1	2.8	6	16.5	0	0.0	2	5.4	1	2.6	1	2.6	23	5.8	1.2
50+	1	9.8	0	0.0	0	0.0	0	0.0	0	0.0	4	33.2	2	16.9	0	0.0	0	0.0	0	0.0	2	15.1	9	7.0	0.5

	Race																								

White, non-Hispanic	41	4.6	50	5.7	59	6.6	58	6.4	56	6.1	58	6.5	46	5.2	40	4.5	46	5.1	51	5.5	68	7.4	573	5.8	29.2
Black non-Hispanic	83	30.4	101	36.8	90	32.9	82	30.5	84	32.3	79	32.2	92	39.1	87	37.9	103	44.8	119	51.3	110	47.2	1030	37.4	52.5
Hispanic	16	12.4	16	12.5	10	7.4	8	5.7	15	10.5	18	12.5	20	13.8	22	15.0	39	26.0	21	13.8	29	18.8	214	13.7	10.9
Other	9	10.9	6	7.2	3	3.4	8	8.8	6	6.2	6	6.1	11	10.9	11	10.6	15	14.1	15	13.4	18	15.2	108	10.0	5.5
Unknown	1	3.5	3	10.0	4	12.2	5	14.3	7	19.7	3	8.8	1	3.0	2	6.4	5	17.0	2	7.1	3	10.6	36	10.4	1.8

	Rank																								

Junior enlisted	86	13.7	101	16.1	68	10.7	77	12.0	88	13.7	77	12.7	84	14.1	81	13.5	96	15.6	118	18.6	114	17.8	990	14.4	50.5
Noncommissioned officer	59	10.8	65	11.9	75	13.3	68	11.8	62	10.7	75	13.1	73	12.8	69	12.2	99	17.3	72	12.5	95	16.5	812	13.0	41.4
Warrant officer	1	6.1	1	6.1	2	11.9	1	5.8	0	0.0	1	5.8	0	0.0	1	5.3	1	5.1	3	14.8	4	19.2	15	7.6	0.8
Commissioned officer	4	1.9	9	4.4	21	10.0	15	6.9	18	8.2	11	5.1	13	6.1	11	5.2	12	5.7	15	7.0	15	6.8	144	6.1	7.3

	Educational level																								

No high school	2	14.6	2	19.7	2	18.2	3	27.4	5	46.3	2	20.0	1	11.0	3	36.0	4	49.5	2	23.0	0	0.0	26	23.9	1.3
High school	130	15.2	148	14.6	129	12.6	120	11.6	126	12.1	132	13.0	140	14.0	130	13.0	168	16.6	175	17.1	192	18.8	1590	14.4	81.1
Some college or more	13	2.7	19	6.0	30	9.2	27	8.0	29	8.4	22	6.2	22	6.3	27	7.3	35	9.3	28	7.3	33	8.5	285	7.1	14.5
Unknown	5	9.2	7	13.8	5	7.6	11	16.5	8	13.9	8	21.2	7	17.7	2	8.0	1	4.0	3	11.1	3	7.9	60	12.3	3.1

	Marital status																								

Married	49	6.5	48	6.6	53	7.2	36	4.7	52	6.8	53	6.9	50	6.5	52	6.7	71	9.0	57	7.1	67	8.2	588	6.9	30.0
Single	95	16.2	126	20.6	109	17.3	119	18.8	114	18.0	108	18.3	112	19.6	101	17.9	131	22.9	146	25.4	151	26.5	1312	20.1	66.9
Other	5	9.8	2	4.0	4	8.0	6	11.9	1	2.0	3	5.8	8	14.8	9	15.9	6	10.0	5	7.8	10	15.1	59	9.7	3.0
Unknown	1	26.2	0	0.0	0	0.0	0	0.0	1	66.7	0	0.0	0	0.0	0	0.0	0	0.0	0	0.0	0	0.0	2	10.3	0.1

	Service																								

Army	48	10.1	61	12.9	56	11.7	62	12.7	54	11.0	58	11.9	66	13.4	60	11.8	96	18.1	88	16.1	88	15.7	737	13.3	37.6
Navy	71	19.4	92	25.0	90	23.9	79	21.0	91	24.5	82	22 9	79	22.9	72	21.6	87	26.6	83	25.5	72	22.3	898	23.2	45.8
Air Force	4	1.1	5	1.4	3	0.8	1	0.3	2	0.5	6	1.7	4	1.2	6	1.8	4	1.2	8	2.4	44	13.3	87	2.3	4.4
Marine Corps	24	14.0	16	9.3	14	8.l	13	7.4	15	8.5	16	9.0	18	10.1	20	11.0	19	9.8	23	11.3	19	9.4	197	9.8	10.0
Coast Guard	3	8.6	2	5.7	3	8.2	6	15.6	6	15.4	2	5.1	3	7.5	4	9.9	2	4.8	6	14.2	5	11.9	42	9.8	2.1

**Table 2:** Characteristics of unique United States military personnel with ≥1 sexually transmitted infection (STI) diagnosis after human immunodeficiency virus-1 diagnosis.

	1 STI diagnosis		2 STI diagnoses		≥3 STI diagnoses	
	No.	Percent	No.	Percent	No.	Percent
Total	471	100.0	157	100.0	39	100.0

Sex						
Male	459	97.5	155	98.7	38	97.4
Female	12	2.5	2	1.3	1	2.6
Age group						
17–19	10	2.1	2	1.3	2	5.1
20–24	152	32.3	59	37.6	19	48.7
25–29	129	27.4	54	34.4	9	23.1
30–34	71	15.1	23	14.6	6	15.4
35–39	66	14.0	13	8.3	3	7.7
40–44	35	7.4	6	3.8	0	0.0
45–49	5	1.1	0	0.0	0	0.0
50+	3	0.6	0	0.0	0	0.0
Race						
White, non-Hispanic	134	28.5	33	21.0	4	10.3
Black, non-Hispanic	255	54.1	94	59.9	29	74.4
Hispanic	53	11.3	19	12.1	3	7.7
Other	24	5.1	8	5.1	3	7.7
Unknown	5	1.1	3	1.9	0	0.0
Rank						
Enlisted	440	46.7	147	46.8	39	50.0
Junior enlisted	196	41.6	66	42.0	22	56.4
Noncommissioned officer	244	51.8	81	51.6	17	43.6
Warrant officer	3	0.6	1	0.6	0	0.0
Commissioned officer	28	5.9	9	5.7	0	0.0
Education level						
No high school	5	1.1	2	1.3	0	0.0
High school	390	82.8	133	84.7	35	89.7
Some college or more	69	14.6	22	14.0	4	10.3
Unknown	7	1.5	0	0.0	0	0.0
Marital status						
Married	143	30.4	39	24.8	10	25.6
Single	314	66.7	112	71.3	28	71.8
Other	14	3.0	6	3.8	1	2.6
Service						
Army	172	36.5	69	43.9	15	38.5
Navy	233	49.5	65	41.4	18	46.2
Air Force	17	3.6	6	3.8	0	0.0
Marine Corps	40	8.5	15	9.6	5	12.8
Coast Guard	9	1.9	2	1.3	1	2.6

**Table 3:** Characteristics of United States active duty military personnel with sexually transmitted infections after their human immunodeficiency virus-1 diagnosis.

	Chlamydia			Gonorrhea			Syphilis			HSV		HPV	
	No. individuals	Incident cases^a^	Rate (per 100,000 HIV p-yrs)	No. individuals	Incident cases^a^	Rate (per 100,000 HIV p-yrs)	No. individuals	Incident cases^a^	Rate (per 100,000 HIV p-yrs)	Incident cases^a^	Rate (per 100,000 HIV p-yrs)	Incident cases^a^	Rate (per 100,000 HIV p-yrs)

Total	91	110	1,960.0	157	186	3,314.2	312	364	6,485.3	84	1,496.7	213	3,795.3

	Sex												

Male	87	104	1,922.8	153	185	3,420.3	312	364	6,729.7	80	1,479.1	207	3,827.1
Female	4	6	2,949.7	4	1	491.6	0	0	0.0	4	1,966.5	6	2,949.7

	Age group												

17–19	2	3	2,464.5	3	3	2,464.5	7	8	6,571.9	2	1,643.0	6	4,928.9
20–24	40	44	2,348.1	70	79	4,215.8	104	118	6,297.1	26	1,387.5	78	4,162.5
25–29	31	41	2,900.6	45	63	4,457.1	91	108	7,640.7	27	1,910.2	58	4,103.3
30–34	11	14	1,177.2	21	21	1,765.8	45	52	4,372.6	16	1,345.4	35	2,943.1
35–39	7	8	1,124.5	15	18	2,530.2	38	46	6,466.1	11	1,546.2	24	3,373.6
40–44	0	0	0.0	3	2	868.9	21	24	10,427.4	2	868.9	11	4,779.2
45–49	0	0	0.0	0	0	0.0	4	5	8,758.2	0	0.0	1	1,751.6
50+	0	0	0.0	0	0	0.0	2	3	19,675.9	0	0.0	0	0.0

	Race												

White, non-Hispanic	11	15	910.1	25	25	1,516.9	57	64	3,883.2	23	1,395.5	76	4,611.3
Black, non-Hispanic	68	82	2,764.4	103	128	4,315.2	197	232	7,821.2	46	1,550.8	102	3,438.6
Hispanic	9	10	1,666.5	14	19	3,166.4	35	42	6,999.4	10	1,666.5	22	3,666.4
Other	3	3	1,084.2	12	12	4,336.6	19	22	7,950.5	3	1,084.2	11	3,975.3
Unknown	0	0	0.0	3	2	1,652.1	4	4	3,304.2	2	1,652.1	2	1,652.1
Rank													
Enlisted	90	109	2,125.5	152	180	3,509.9	298	347	6,766.4	78	1,521.0	196	3,821.9
Junior enlisted	43	50	2,120.2	85	95	4,028.4	128	142	6,021.5	35	1,484.2	95	4,028.4
Senior enlisted	47	59	2,129.9	67	85	3,068.5	170	205	7,400.5	43	1,552.3	101	3,646.1
Warrant officer	1	1	2,766.4	0	0	0.0	2	4	11,065.7	1	2,766.4	1	2,766.4
Commissioned officer	0	0	0.0	5	6	1,339.9	12	13	2,903.2	5	1,116.6	16	3,573.2

	Educational level												

No high school	0	0	0.0	1	1	1,491.2	3	5	7,455.9	1	1,491.2	1	1,491.2
High school	84	100	2,200.8	142	170	3,741.3	258	299	6,580.3	68	1,496.5	181	3,983.4
College or more	7	10	1,189.1	13	14	1,664.8	48	57	6,778.0	14	1,664.8	29	3,448.5
Unknown	0	0	0.0	1	1	623.5	3	3	1,870.6	1	623.5	2	1,247.1

	Marital status												

Single	25	76	2,017.7	43	133	3,531.0	93	250	6,637.1	60	1,592.9	151	4,008.8
Married	62	29	1,735.4	108	46	2,752.8	211	106	6,343.3	22	1,316.5	56	3,351.2
Other	4	5	2,981.8	6	7	4,174.5	8	8	4,770.8	2	1,192.7	6	3,578.1

	Service												

Army	49	62	2,891.9	56	66	3,078.5	128	143	6,670.1	39	1,819.1	71	3,311.7
Air Force	28	32	1,186.4	72	87	3,225.5	138	169	6,265.5	37	1,371.7	112	4,152.3
Navy	4	6	3,650.4	3	2	1,216.8	12	12	7,300.7	2	1,216.8	7	4,258.8
Marines	9	9	1,832.5	23	28	5,701.2	29	32	6,515.7	4	814.5	18	3,665.1
Coast Guard	1	1	865.3	3	3	2,595.9	5	8	6,922.4	2	1,730.6	5	4,326.5

HSV: herpes simplex virus.

HPV: human papillomavirus.

^a^Each individual was only eligible to be an incident case once per 30-day period for chlamydia and gonorrhea, once per 13-month period for syphilis, and once per lifetime for HSV and HPV.
